# The Burden of Depression and Stigma Among Individuals With Tuberculosis Within the Integrated Tuberculosis-Mental Health Project: A Multisite Mixed-Methods Study Protocol

**DOI:** 10.7759/cureus.88518

**Published:** 2025-07-22

**Authors:** Ngozi Murphy-Okpala, Charles Nwafor, Chinwe Eze, Martin Njoku, Okechukwu Ezeakile, Anthony Meka, Ngozi Ekeke, Francis Iyama, Daniel Egbule, Chukwuma Anyaike, Obioma Chijioke-Akaniro, Joseph Chukwu, Beatrice Kirubi, Jacob Creswell, Justus U Onu

**Affiliations:** 1 Public Health, RedAid Nigeria, Enugu, NGA; 2 Public Health, Federal Ministry of Health and Social Welfare, Nigeria, Abuja, NGA; 3 Public Health, Stop TB Partnership, Abuja, NGA; 4 Mental Health, Nnamdi Azikiwe University, Nnewi, NGA; 5 Psychiatry, Federal Neuropsychiatric Hospital, Enugu, NGA

**Keywords:** burden, depression, dimensions, nigeria, stigma, tuberculosis

## Abstract

There is a complex bidirectional relationship between tuberculosis (TB), major depressive disorder (MDD), and stigma. Despite the significant burden of these conditions in sub-Saharan Africa, only a few studies have explored their interplay. The primary aim of this study is to determine the burden of MDD and the dimensions of stigma among individuals with TB. The secondary aim is to assess the effectiveness of integrated TB-depression treatment compared to standard TB treatment. This will be a multistage study utilizing a mixed-methods design to address the research questions. Stage 1 will use a cross-sectional design to evaluate the burden of depression and stigma. Depression will be assessed using the Patient Health Questionnaire-9 (PHQ-9), and stigma will be measured using Van Rie’s TB stigma scales - covering self-stigma, secondary stigma, community stigma, and stigma among healthcare workers. Stage 2 will involve a longitudinal follow-up of all eligible participants diagnosed with both TB and MDD. Participants will receive either integrated TB and MDD treatment or standard TB treatment, depending on their assigned site. Baseline assessments will include depressive symptomatology (PHQ-9) and perceived social support (Oslo Social Support Scale, OSSS). Follow-up assessments will occur at two weeks, eight weeks, and 24 weeks, using the same instruments. TB-related outcomes - including treatment continuation, interruption, default, and mortality - will also be recorded. Stage 3 will use a qualitative approach to explore the experience and dimensions of stigma from the perspectives of service users, their family members, and their communities. Weighted prevalence of MDD will be estimated with 95% CIs. The proportion of participants reporting experiences of stigma will be described using frequency counts and percentages. Changes in depressive symptoms over time between the two treatment groups will be analyzed using mixed ANOVA. Qualitative data will be analyzed thematically.

## Introduction

Tuberculosis (TB) remains a leading cause of morbidity and mortality globally [1]. In 2022, an estimated 10.6 million people developed TB, with nearly a quarter of cases occurring in Africa, where the disease led to approximately 26 deaths per 100,000 population [1]. The burden of TB is further compounded by its frequent comorbidity with depression and the high levels of associated stigma [2,3].

Depression is a serious mental disorder projected to become the leading contributor to the global burden of disease by 2030 [4]. There is a high comorbidity between TB and major depressive disorder (MDD) [5]. A recent meta-analysis in Africa reported that approximately four out of every 10 individuals with TB also suffer from MDD [6], highlighting significant implications for TB control programs in sub-Saharan Africa. Comorbid depression among persons with TB has been associated with delayed health-seeking behavior and poor treatment adherence, which in turn contribute to higher morbidity and mortality, drug resistance, and continued transmission of the disease [6]. Identified risk factors for MDD in individuals with TB include female gender, poor social support, low educational attainment, rural residence, high internalized stigma, and clinical symptoms such as dyspnea [7].

Similarly, TB-related stigma is widespread in the region, with nearly half of TB patients in Africa reporting perceived stigma [8]. This stigmatization is often rooted in misconceptions about TB’s contagiousness, limited public knowledge about its transmission, and its association with HIV [9]. Stigma contributes to psychological distress and poses a significant barrier to effective public health interventions aimed at TB prevention and control [10]. Its effects mirror those of depression, particularly in delaying diagnosis and hindering treatment adherence [8]. Both stigma and MDD independently impact TB outcomes by compromising timely diagnosis and effective treatment.

A complex bidirectional relationship exists between TB and depression [5]. TB infection or reactivation, as well as its treatment, may trigger depressive symptoms through biological (e.g., malnutrition, inflammatory responses, hypothalamic-pituitary-adrenal axis dysregulation, and medications like isoniazid or cycloserine), behavioral (e.g., substance use), and social mechanisms (e.g., stigma, financial hardship, and marital difficulties) [5]. Conversely, depression may worsen TB outcomes by contributing to immunosuppression, increased inflammatory responses, and altered metabolic pathways. Furthermore, depression can negatively affect treatment adherence and delay health-seeking behavior, reducing access to care and increasing disease burden [5].

This intricate interplay between TB and MDD has important treatment implications. It has been hypothesized that adjunctive antidepressant therapy could enhance treatment outcomes in patients with TB [5]. Conversely, it is also believed that treating TB alone may help alleviate depressive symptoms in those with comorbid depression [5]. The latter forms the secondary focus of this study, which builds upon an ongoing project by RedAid Nigeria that integrates care for TB, depression, and substance use disorder (SUD) in clinical settings.

RedAid Nigeria is currently implementing the TBR W10 Project, an integrated service delivery model designed to expand access to person-centered care for TB, mental health (MH), and SUDs across 10 intervention local government areas (LGAs) in three states in Southeast and North-central Nigeria. As findings from this pilot initiative are expected to inform future policy and potential scale-up, it is critical to assess and document TB-related stigma among persons with TB and those affected by MH/SUD, as well as among other stakeholders. Such assessment will help elucidate the social and contextual factors influencing health-seeking behaviors and service delivery among TB patients. The current protocol is a nested study within the TBR W10 project and aims to quantify the burden of depression and stigma among individuals with TB.

Aim

The primary aim is to determine the burden of depression and stigma among persons with TB and to explore the understanding of TB-related stigma at the individual, family, and community levels across different settings. The secondary aim is to evaluate the effectiveness of integrated TB and depression care compared to standard TB care in reducing depressive symptoms over the course of treatment follow-up.

Objectives

The objective of the study is to determine the prevalence of MDD and the level and dimensions of stigma among persons with TB, explore how TB-related stigma is understood across different stakeholder groups, and assess the effectiveness of integrated TB and depression care compared to standard TB care in reducing depressive symptoms over time.

## Materials and methods

Study design and setting

This study will adopt a mixed-methods design comprising both quantitative and qualitative components, embedded within the ongoing TB REACH Wave 10 (TBRW10) project. The project is being implemented in three Nigerian states - Anambra and Enugu in southern Nigeria and Nasarawa in northern Nigeria - between July 2023 and August 2024, with a no-cost extension through December 2024.

The study will be conducted in the TB REACH W10 intervention LGAs, which include Awka North, Awka South, Nnewi North, and Onitsha South in Anambra State, and Awgu, Enugu East, Enugu North, and Igboeze North in Enugu State. The control sites will include Nasarawa Eggon and Keffi LGAs in Nasarawa State.

In the intervention areas, screening officers based in selected public and private primary, secondary, and tertiary health facilities, along with community health workers, were trained to conduct integrated screening for TB, depression, and SUDs using a TB symptom checklist, Patient Health Questionnaire-2 (PHQ-2)/Patient Health Questionnaire-9 (PHQ-9), and the ASSIST tool, respectively. Individuals with presumptive TB are guided through sputum sample collection - or chest X-ray, where indicated - for GeneXpert MTB/RIF testing. Those diagnosed with co-morbid depression or SUD are offered psychoeducational counseling.

TB diagnosis in both intervention and control areas follows the standard WHO guidelines as adapted in Nigeria's national TB guidelines. Diagnosed TB cases may be bacteriologically confirmed (i.e., at least one positive sputum sample via acid-fast bacillus (AFB) smear or GeneXpert MTB/RIF test) or clinically diagnosed - defined as negative AFB or GeneXpert results in the presence of clinical symptoms and suggestive chest radiographic findings, as determined by the attending physician. Across all project facilities, an average of 299 TB cases are diagnosed monthly, with the project directly contributing an average of 87 cases per month.

In the control arm, health facilities continue to provide standard-of-care services, with TB, depression, and SUD services offered separately. Presumptive TB cases are guided on sputum collection (or chest X-ray, when necessary) for GeneXpert MTB/RIF testing. Patients identified with depressive symptoms or SUDs are referred to the nearest facility equipped for MH services. Across control facilities, an average of 188 TB cases are diagnosed each month.

The control LGAs were selected based on their similarity to the intervention areas in terms of demographic and epidemiological profiles. Importantly, these control LGAs are geographically non-contiguous with any of the intervention LGAs, helping to minimize contamination. Both groups share comparable population characteristics.

Ethical considerations

Ethical approval for this study was obtained from the Health Research and Ethics Committee of the University of Nigeria Teaching Hospital, Enugu, Nigeria (reference no. UNTH/HREC/2024/09/2127). The study will adhere strictly to international ethical norms and standards. Written informed consent will be obtained from all participants. They will be clearly informed of their right to withdraw from the study at any point, even after providing initial consent, without any impact on their medical care.

Participants will also be informed that if any previously undiagnosed clinical condition is identified during the course of the study, it will be discussed with them, and appropriate medical information - including referral options - will be provided. All data collected will be stored on an encrypted computer, accessible only to the research team, to ensure confidentiality and data security.

Quantitative arm

This component is conceptualized in two interconnected stages (Stages 1 and 2), which are not mutually exclusive.

Stage 1

Design: A cross-sectional design will be employed to determine the burden of MDD, the levels and dimensions of stigma, and selected psychometric properties of stigma measurement tools in our study setting.

Sample size and sampling technique: The sample size calculation is based on the primary objective of quantifying the burden of depression and stigma among persons with TB. Using a pooled prevalence estimate of depression in persons with TB of 39.4% [6], a 95% CI, a 5% margin of error, and an estimated monthly population of 299 newly diagnosed TB cases within the project, a minimum sample size of 165 is required. This sample size will also be sufficient for assessing the psychometric properties of the stigma questionnaires, as it exceeds the requirement for factor analysis - typically between five and seven participants per item [11]. For example, the Van Rie Self-Stigma Scale, which contains 12 items, would require a minimum of 84 participants (7 × 12) for psychometric evaluation, making the planned sample size adequate.

Although the sample size is not specifically powered for the secondary outcome - assessing the effectiveness of the one-stop-shop integrated TB and depression treatment compared to treatment as usual - it is anticipated that all eligible participants per arm will provide sufficient data for meaningful comparative analysis.

Participants will be recruited consecutively as they present to the study sites, provided they meet the inclusion criteria. Eligibility for depression screening includes being newly diagnosed with TB, being 18 years or older, and not having chronic medical conditions such as HIV or cancer. To assess stigma, the study will also include family members, healthcare workers involved in TB care, and select willing community members.

Data collection: Data collection will occur in two phases and will involve all newly diagnosed TB patients at the study sites. In Phase 1, all diagnosed participants will be initially screened for depression using the PHQ-2. A cutoff score of 3 or above will indicate a positive screen for depression. Participants who screen positive on the PHQ-2 will subsequently be assessed using the PHQ-9 to determine whether they meet the diagnostic criteria for MDD. Additionally, 20% of participants who screen negative on the PHQ-2 will also be evaluated using the PHQ-9 to strengthen diagnostic validity. TB-related stigma - across self, family (secondary), community, and healthcare workers - will be assessed using the corresponding stigma scales designed for each domain.

Study questionnaires: The PHQ-2 is a 2-item depression screening tool rated on a 4-point Likert scale. It comprises the first two questions of the PHQ-9 and has shown modest validity indices as a screening instrument [12]. At a cutoff score of ≥3, the PHQ-2 has demonstrated sensitivity of 82.9%, specificity of 90%, and a positive predictive value of 34.9% [12]. The total score ranges from 0 to 6.

The PHQ-9 is a 9-item, multipurpose instrument used to screen, diagnose, monitor, and assess the severity of depression. Initially developed to improve depression detection in primary care and non-psychiatric settings [13], the PHQ-9 uses a 4-point Likert scale (0-3) and yields a total score ranging from 0 to 27. Scores of 5-9 suggest mild depressive symptoms; 10-14, moderate; 15-19, moderately severe; and ≥20, severe depression [13]. Despite its brevity, the PHQ-9 has demonstrated strong psychometric properties [13,14]. A PHQ-9 score of ≥10 yields both sensitivity and specificity of 88% for MDD [13].

The stigma scales are TB-specific tools designed to evaluate different dimensions of stigma, including self-stigma, family (secondary), community, and healthcare workers’ attitudes. Each scale uses a 5-point Likert response format and has shown modest psychometric strength [15].

Questionnaire translation: A two-stage translation process will be used to translate the PHQ-2, PHQ-9, and the stigma scales - covering self, family (i.e., secondary), community, and healthcare workers - into Igbo and Hausa. In the first stage, two bilingual researchers will independently translate the tools from English into the respective local languages. In the second stage, these translated versions will be back-translated into English. The research team will then convene a consensus meeting to review and harmonize both the translations and back-translations to ensure accuracy and cultural relevance. Participants who do not understand English will complete the version translated into their local language.

Data analysis: Data will be analyzed using IBM SPSS Statistics for Windows, Version 20.0 (Released 2011; IBM Corp., Armonk, NY, USA). Weighted prevalence will be calculated with 95% CIs. The severity of depression will be categorized based on PHQ-9 scores as follows: mild (5-9), moderate (10-14), moderately severe (15-19), and severe (20-27), and will be described using frequency counts and percentages. The burden of self, family, community, and healthcare workers’ stigma will be assessed using established cutoff scores. Summary statistics, including medians and interquartile ranges, will be used to describe item-level scores. Exploratory factor analysis will be conducted to identify the dimensions of stigma within the Nigerian context. The internal consistency of the stigma scales will be assessed using Cronbach’s alpha.

Stage 2

Design: A quasi-experimental design will be used to evaluate the effectiveness of integrated, one-stop treatment for TB and MH conditions on depressive symptoms over time, compared with the standard TB treatment approach (i.e., anti-TB medications with referral to other health facilities for management of depressive symptoms).

Procedure: Participants diagnosed with MDD at the participating sites will form the sample population for this stage. The program currently includes 12 sites across three states. Of these, 10 sites have been designated as the intervention arm, where integrated TB and common mental disorder treatment is being piloted. The remaining two sites serve as the comparative arm, where TB treatment is provided but MH comorbidities are referred to external facilities.

This stage will leverage the existing infrastructure to evaluate the effectiveness of integrated TB and MH treatment compared to treatment as usual. Baseline assessments will include depressive symptomatology and social support, measured using the PHQ-9 and the Oslo Social Support Scale (OSSS), respectively. The OSSS is a brief self-report tool comprising three items that assess support from close confidants, a sense of concern from others, and access to practical support. It is scored on a 5-point Likert scale, with total scores ranging from 3 to 15.

Social support, sociodemographic variables (e.g., age, sex, marital status, and educational level), and clinical characteristics (e.g., dyspnea assessed through clinical history) will be measured and considered as potential covariates. Follow-up assessments will take place at two weeks, eight weeks, and 24 weeks. These intervals correspond to key phases in TB treatment - onset of symptom relief, active treatment phase, and continuation phase. The baseline instruments (PHQ-9 and OSSS) will be re-administered at each follow-up point. Additionally, TB-related outcomes (e.g., treatment continuation, interruption, default, and death) will be recorded.

Participants aged 18 years and older, diagnosed with TB and comorbid depression, will be eligible for inclusion upon providing informed consent. Exclusion criteria will include individuals with suicidal plans or attempts, those presenting with psychotic symptoms, those planning to transfer TB care to another facility, those who are too ill to participate, and those with other comorbid chronic conditions. Participants will be recruited consecutively as they present at the study sites.

Data analysis: Data will be analyzed using IBM SPSS Statistics for Windows, Version 20.0. The normality of distribution for continuous variables will be assessed using the Shapiro-Wilk test. Changes in depressive symptoms (treated as a continuous variable) between the two study arms across the four follow-up intervals will be compared using a mixed ANOVA model, adjusting for covariates such as social support and age. Treatment effects will be estimated between subjects using effect size at each follow-up point.

Multivariate logistic regression analysis will be employed to identify independent predictors of depression (present/absent) at baseline and at six months. Predictors will include age, gender, area of residence, marital status, level of education, and social support. All statistical tests will be two-tailed, with a 95% CI, and a p-value of less than 0.05 will be considered statistically significant.

Qualitative arm

An exploratory phenomenological approach will be used to understand the experience of stigma among persons with TB. This arm will involve in-depth interviews with patients, their family members, and key individuals in the community. Purposive sampling will be used to select two participants from each category per site, ensuring diversity in terms of age, gender, and other relevant characteristics.

Data analysis: Audio recordings will be transcribed verbatim and analyzed thematically, following the guidelines recommended by Braun and Clarke [16]. The lead investigator will carefully read the transcripts, and relevant text segments will be coded, synthesized, and grouped into categories based on similarities in meaning. A combination of deductive and inductive approaches will be used to develop the codes. Initial codes will be informed by predetermined topics from the interview guide and relevant theoretical constructs (deductive approach), while new codes will emerge from the data itself (inductive approach). Coding will continue until no new concepts arise.

To ensure trustworthiness, one of the co-investigators will check for coding consistency. Final themes will be confirmed through a consensus meeting with the research team. To maintain reflexivity, investigators involved in the care of persons with TB will remain aware of and transparent about how their personal experiences and perspectives may influence the analysis and interpretation of the data.

NVivo software will be used to manage and organize the data. The analysis will focus on identifying key themes related to the dimensions of TB-related stigma and its impact on health-seeking behavior among participants and their families. Themes will be interpreted using a contextualist approach, considering the characteristics of the study setting to better understand participants’ experiences. Illustrative quotes will be selected by the principal investigator to exemplify each theme.

## Results

The results of this study will be aligned with the stated objectives. Data will be analyzed using appropriate statistical methods and presented in tables, figures, and descriptive prose, as appropriate.

## Discussion

While this study has several strengths, certain limitations are anticipated. First, the PHQ-9 is primarily a screening tool for MDD; the use of structured diagnostic instruments - such as the Mini-International Neuropsychiatric Interview or the Composite International Diagnostic Interview - would have provided a more definitive diagnosis. In addition, although a wide range of stakeholders will be interviewed to explore perspectives on TB-related stigma, the views of policymakers, law enforcement officials, and media representatives are not included. Their inclusion could have provided a broader and more nuanced understanding of TB stigma from a systemic and sociocultural standpoint.

## Conclusions

The study’s findings will inform the conclusions and recommendations drawn from the data. These results are expected to contribute to evidence-based strategies for integrating MH care into TB services. Future research directions will also be proposed to build on the insights gained from this study.
